# Psychotropic medication use among adolescents participating in three randomized trials of DBT

**DOI:** 10.1186/s40479-024-00249-0

**Published:** 2024-02-22

**Authors:** Lars Mehlum, Joan Asarnow, Sudan Prasad Neupane, Pilar Santamarina-Perez, Mireia Primé-Tous, Gabrielle A. Carlson

**Affiliations:** 1 Institute of Clinical Medicine, Faculty of Medicine, National Centre for Suicide Research and Prevention, University of Oslo, Oslo, Norway; 2 UCLA Center for Youth Suicide & Self-Harm Treatment & Prevention, David Geffen School of Medicine, UCLA, Los Angeles, USA; 3 Department of Child and Adolescent Psychiatry and Psychology, Institute of Neuroscience, Hospital Clínic de Barcelona, 2017SGR88 Barcelona, Spain; 4 Division of Child and Adolescent Psychiatry, Renaissance School of Medicine at Stony Brook University, Stony Brook, NY USA

**Keywords:** Suicide, Self-harm, Adolescent, Dialectical behaviour therapy, Psychotropic medication

## Abstract

**Background:**

Frequently presenting with symptoms of mood or anxiety disorders, substance abuse or borderline personality disorder, suicidal and self-harming adolescents often are prescribed psychotropic medication. Though such treatment may be warranted, recurrent suicidal and self-harming behaviour is often linked to emotion dysregulation where pharmacological treatment has weak empirical support. There is a need for more clinical research into the frequency, type and rationale for pharmacological treatment in this group. In this secondary analysis of three randomized clinical trials of dialectical behaviour therapy for adolescents, we report on psychotropic medication use in the respective samples at the time of recruitment, compare use of psychotropic medication across trials and describe sample characteristics that may be associated with possible differences in psychotropic medication.

**Findings:**

Trials were conducted in Norway, the US and Spain (labelled the Oslo, US and Barcelona samples). At baseline, 86% of the Barcelona sample, 67% of the US sample and 12% of the Oslo sample were taking at least one psychotropic medication with antidepressants as the most frequent, followed by antipsychotics (72%, 22% and 1.3% respectively) and mood stabilizers (14.2%, 16.2% and 0%). In the Oslo sample there was a significant association between receiving a diagnosis of major depression and the likelihood of receiving antidepressants, but no such association was found in the Barcelona and US samples. The overall 7–8 times higher proportion of participants in the US and Barcelona samples treated with psychotropic medication could only partially be explained by differences between the samples in diagnostic profiles, symptom severity or level of dysfunction.

**Conclusions:**

Highly prevalent in use among suicidal and self-harming adolescents with borderline features, psychotropic medication was still very unevenly prescribed across trials, differences not explained by differences in sample characteristics suggesting that current treatment practices are not fully empirically supported. We call for continued medical education and increased availability of evidence-based psychosocial interventions.

**Supplementary Information:**

The online version contains supplementary material available at 10.1186/s40479-024-00249-0.

## Background

Adolescent suicide, suicide attempt, suicidal ideation, and non-suicidal self-injury (NSSI) are major public health concerns worldwide. In Europe and North America, suicide is the second leading cause of death among young people between 10 and 19 years [1]. The lifetime prevalence of suicide attempts ranges from 4.6% in Europe to 8.6% in North America. Approximately 18.5% of children and adolescents report lifetime NSSI [2]. For the age group 10–19 years, suicide mortality per 100,000 population in the US, Norway and Spain are 5.9, 3.0 and 1.5 respectively [1].

Adolescents who engage in self-harming behaviours present with a varying degree of psychiatric morbidities, including depression, anxiety, borderline personality disorder (BPD), eating disorders and substance use disorders. Many youths with self-harming behaviours receive pharmacotherapy, usually in the context of depressive disorders. Pharmacotherapy may indeed be indicated in unstable borderline patients to address symptoms of anxiety or thought disturbances in psychosis. Whereas suicide risk may be reduced through pharmacotherapy in conditions such as bipolar disorder, schizophrenia and depression, empirical support for such effects is, however, lacking for the use of pharmacotherapy in younger borderline patients with self-harming behaviours. In fact, studies suggest that some SSRIs may increase suicidal thoughts and behaviours during the first few months of their use in young people [3]. A number of studies have shown strongly rising trends in prescribing of psychotropic medication to adolescents in Europe [4, 5] and North-America [6], although trends have been stabilizing in the US in recent years on a relatively high level. We have previously raised concerns about the widespread use of psychopharmacotherapy, sometimes in the form of polypharmacy, among self-harming adolescents and we have called for increased research in this area [7]. This is even more important considering emerging evidence from clinical trials suggesting that psychosocial interventions, such as dialectical behaviour therapy (DBT) and some forms of cognitive-behaviour therapy and mentalization-based therapy are effective in reducing self-harm behaviours among adolescents [8].

## Methods

The authors of this brief report include investigators from the three [9–11] randomized clinical trials of DBT with adolescents presenting with suicidal ideation and self-harm behaviour published to date. These trials were conducted in Norway [9], the US [10] and Spain [11] (referred hereafter to as the Oslo, US and Barcelona samples) (inclusion and exclusion criteria in the three trials are shown in Supplementary Table 2). DBT, originally developed by Linehan for adults with repetitive suicidal behaviours in the context of BPD, and later adapted for use with adolescents (e.g., including families in skills training groups), focuses on helping patients improve their emotion regulation capacity, distress tolerance and interpersonal effectiveness through individual, group-based, and family therapy sessions supplemented with phone coaching as needed. Meta-analysis suggests a clear improvement in suicidal ideation and self-harm measures post-intervention with DBT [12]. Further analyses show that non-response is limited to a small proportion of the treated samples (13%) [13], and that DBT is associated with improved treatment adherence and emotion regulation and reduced depression and hopelessness symptoms.

In this secondary analysis of the three trial data sets we report on psychotropic medication use in the respective samples at the time of recruitment, compare use of psychotropic medication across trials and describe sample characteristics that may be associated with possible differences in psychotropic medication. We identified drugs used for psychiatric treatment (yes/no) and classified each drug into the major categories reported in Table 2 based on an online pharmaceutical encyclopaedia (www.drugs.com). In all trials, psychiatric assessment was performed by trained clinician-researchers using the Schedule for Affective Disorders and Schizophrenia for School-Aged Children (KSADS). All trial participants reported recent and repetitive self-harm behaviour, and samples were otherwise fairly comparable in terms of age (≈ 15 years), gender (≈ 90% female), ethnicity (predominantly Caucasian), socio-economic status and recruitment procedures (Table 1). All participants and parents provided written informed consent before inclusion. The original studies are registered in ClinicalTrials.gov/; NCT00675129 (Oslo); NCT01528020 (US); NCT02406625 (Barcelona) and methodological details are reported in respective publications [9–11].

**Table 1:** Baseline sociodemographic and clinical characteristics of the trial samples of the three published randomized controlled trials of DBT-A for suicidal and self-harming adolescents with borderline features

Characteristic	US sample (*N* = 173)		Oslo sample (*N* = 77)		Barcelona sample (*N* = 35)	
	*n*	*%*	*n*	*%*	*n*	*%*
*Sociodemographic characteristics*						
Female sex	163	94.8	68	88.3	31	88.6
Age, mean (SD) years	14.9	1.5	15.6	1.5	15.0	1.4
Parents currently co-habiting	82	54.7	34	44.2	14	40.0
Race/ethnicity						
Caucasian/White	97	56.4	66	85.7	26	74.3
Latina	48	27.5	3	3.9	5	14.3
Black/African American	12	7.0	0	0	1	2.8
Asian	10	5.9	5	6.5	2	5.7
Other	5	2.9	3	3.9	1	2.8
Family annual income						
< 15 000 $ or € Equivalent	15	11.0	-	-	6	17.2
15 000–29 999	9	6.6	-	-	4	11.4
30 000–49 999	25	17.4	-	-	9	25.7
≥ 50 000	95	65.3	-	-	16	45.7
*Clinical characteristics* Current psychiatric diagnosis						
Any depressive disorder	145	83.8	46	59.7	29	82.9
Major depressive disorder	142	82.1	17	22.1	29	82.9
Bipolar disorders	-	-	0	0	5	14.2
Any anxiety disorder	93	54.1	33	42.9	19	54.3
Posttraumatic stress disorder	78	45.1	13	16.9	8	22.9
Any substance use disorder	-	-	2	2.6	15	42.9
Eating disorder	1	0.7	6	7.8	18	51.4
Borderline Personality disorder	92^c^	53.2	15^c^	20.5	-	-
ADHD	-	-	4	5.2	11	31.4
*Self-harm and related variables*						
Suicidal ideation mean (SD)^a^	47.6	17.4	36.9	23.7	45.2	17.1
Any lifetime NSSI (l%)	163	94.2	77	100	35	100
Lifetime number of NSSI (SD)	26.3	47.2	34.0	88.0	-	-
Any Lifetime Suicide attempt (%)^b^	173	100	26	38	26	74.3
Number of SA (lifetime) mean (SD)	-	-	1.7	4.2	1.9	1.8
Level of functioning (C-GAS) mean (SD)	45.2	7.4	56.1	8.3	53.1	6.4

Notes: All diagnoses made by the *KSADS*, NSSI = non-suicidal self-injury; SA = suicide attempt; ^a^ = as assessed by the *Suicidal Ideation Questionnaire- Jr*; ^b^ = calculated % is for non-missing data only (Oslo sample) and lifetime data; ^c^ ≥ 3 Borderline Personality Disorder traits required for study inclusion, “- " Denotes non-available information

## Results

At baseline, 86% of participants of the Barcelona sample, 67% of the US sample and 12% of the Oslo sample were taking at least one psychotropic medication (Table 2). Antidepressants were most commonly used; among them selective serotonin reuptake inhibitors (SSRI) were the most commonly prescribed; 73% of all participants on any psychotropic medication used at least one SSRI. Because psychotropic prescriptions could presumably be given to treat depressive disorders, we disaggregated the participants with and without major depression. In the US and Barcelona samples, the subsample of participants who had current depression did not have a higher rate of psychotropic medication use than the full samples. However, in the Oslo sample three times as many participants with major depression received psychotropic medication compared to the full sample. While 35% of participants with major depression in the Oslo sample used any psychotropic medication, the rates were 86% in the Barcelona sample and 65% in the US sample (Table 2). In order of frequencies, other drugs used by the US sample were antipsychotics (22%), mood stabilizers (16%); by the Barcelona sample were antipsychotics (72%), anxiolytics or CNS stimulants (17% each); whereas less than 3% of the Oslo sample used any of these. The data did not provide direct evidence of polypharmacy in any of the study samples. The mean number of psychotropic medications was 1.44 (SD 1.5) in the US sample, 1.8 (SD 1.13) in the Barcelona sample and 0.13 (SD 0.38) in the Oslo sample. More detailed data on use of psychotropic medication in the samples are reported in Supplementary Table 1.

**Table 2:** Baseline use of psychotropic medications across samples of the three published randomized controlled trials of DBT-A for suicidal and self-harming adolescents with borderline features

Drug class	US sample					Oslo sample					Barcelona sample			
	All participants (*N* = 173)		Participants with MD (*n* = 142; 82.1%)			All participants (*N* = 77)		Participants with MD (*n* = 17; 22.1%)			All participants (*N* = 35)		Participants with MD (*n* = 29; 82.9%)	
	*n*	*%*	*n*	*%*		*N*	*%*	*n*	*%*		*n*	*%*	*n*	*%*
Any medication with psychiatric use	116	67.1	92	64.8		9	11.7	6	35.3		30	85.7	25	86.2
Antidepressant (any)	104	60.1	82	57.7		5	6.5	2	11.8		23	65.7	21	72.4
SSRI	90	52.0	71	50.0		4	5.2	1	5.9		19	54.3	-	-
SNRI	8	4.6	6	4.2		0	0	0	0		2	5.7	-	-
Tricyclic	1	0.6	1	0.7		0	0	0	0		1	2.8	-	-
Other	22	12.7	18	12.7		1	1.3	1	5.9		1	2.8	-	-
Anxiolytic (any)	13	7.5	12	8.5		0	0	0	0		0	0	5	17.2
Benzodiazepine	10	5.8	10	7.0		0	0	0	0		5	14.3	-	-
Antipsychotic	38	22.0	30	21.1		1	1.3	1	5.9		26	74.2	21	72.4
Mood stabilizer ^a^	28	16.2	24	16.9		0	0	0	0		5	14.2	4	13.8
CNS Stimulant	17	9.8	13	9.2		1	1.3	1	5.9		5	14.2	5	17.2
Alpha adrenergic	5	2.9	4	2.8		0	0	0	0		0	0	-	-
Antihistamine	8	4.6	8	5.6		2	2.6	2	11.8		0	0	-	-
Other ^b^	3	1.7	2	1.4		1	1.3	0	0		1	2.8	1	3.4
Total number of medications (mean, SD)	1.44	1.51	1.44	1.56		0.13	0.38	0.16	0.36		1.8	1.13	-	-

Notes: MD = major depression; SSRI = selective serotonin reuptake inhibitors; SNRI = serotonin and norepinephrine reuptake inhibitors; ^a^ the category ‘Mood stabilizer’ includes lithium and/or anticonvulsant; ^b^ other medications include anticholinergic, melatonin, beta blocker or any other medication used for psychiatric purpose. “- " denotes non-available information

As shown in Table 1, the Oslo sample had fewer participants satisfying diagnostic criteria for any depressive disorder compared to the US and Barcelona samples; this was most notable for major depression where differences were large (22% vs. 82%). Non-suicidal self-injury was reported by nearly all participants across samples with the highest life-time number of episodes in the Oslo sample, while the US and Barcelona samples had the highest proportions of participants with any life-time suicide attempts, and they reported higher levels of suicidal ideation. Participants of the US sample had the lowest average level of functioning (C-GAS).

## Discussion

This cross-national comparison showed that psychotropic medication was used by the majority of US and Barcelona, but not Oslo adolescents at the time of their recruitment to these treatment trials. Participants in the Oslo sample had a somewhat less severe diagnostic profile, symptom severity and level of dysfunction, however, the substantial difference between trials, such as a 7–8 times higher proportion of participants in the US and Barcelona samples compared to the Oslo sample used psychotropic medication, could only partially be explained by these sample differences. The findings should be interpreted in light of some study limitations: Our clinical trials were not designed to study which indications that were used for psychotropic medication prescription in each participant or whether medication use changed the course of treatment response. Furthermore, our trials were unable to evaluate whether medication use changed treatment response. Finally, the Barcelona sample consisted of significantly fewer participants which makes the figures more uncertain. However, the widespread use of psychotropic medication in these samples of suicidal and self-harming adolescents with borderline features suggests that prescriptions may not always have adhered to evidence-based guidelines, but possibly were prompted by the need to “do something” in the absence of better alternatives. In many countries psychotherapy is a very limited resource and too expensive for many families, thus making it an unrealistic treatment alternative for patients without access [14]. In this context, the comparatively more accessible mental health care for people under 18 in Norway may be an exception to this rule, possibly allowing for earlier referrals (thus the somewhat less severe symptom profile in the Oslo sample) and less need for psychotropic medication at such an early stage in life. The highly prevalent use of medication in our samples could possibly also be associated with a relative lack amongst many physicians or psychiatrists of updated knowledge on evidence based psychotherapeutic interventions having been developed over the past 1–2 decades. To change routine clinical practice is very time consuming and requires substantial resources for dissemination, training, and implementation. Developers of novel and evidence-based psychosocial interventions within child and adolescent mental health care are, however, in no way able to match marketing budgets of the pharmaceutical industry. Health systems and policy makers could do more to promote systematic implementation of treatments with evidence of effectiveness on suicidal and self-harming behaviours in adolescents, an approach consistent with the Zero Suicide framework implemented in an increasing number of systems within the United States. DBT adapted for suicidal and self-harming adolescents does not only aim to prevent suicide and self-harm behaviour but aims to help participants change problem behaviours and coping strategies associated with personality dysfunction to get a life worth living instead of embarking on a long-term trajectory of dysfunction.

In this brief report we have only considered what psychotropic medication participants were using at the time of their recruitment, not what medication they were using during the treatment trial or whether medication enhanced or detracted from treatment response. It is also important to remember that individuals recruited to participate in RCTs, such as ours, may not be fully representative of the broader clinical population; thus limiting the generalizability of our findings. Although it falls beyond the scope of our study, possible interactions between psychotherapy and psychotropic medication in this vulnerable population merits, however, considerable interest and is an important focus for future studies.

## Conclusions

In conclusion, our data show that psychotropic medication use was highly prevalent in different settings among adolescents with suicidal and self-harming behaviour and borderline features despite this practice not necessarily being empirically supported. We call for continued medical education and increased availability of evidence-based psychosocial interventions. Further research should examine potential interactions between psychotropic medications and psychotherapy in this population.

### Electronic supplementary material

Below is the link to the electronic supplementary material.

Supplementary Material 1

Supplementary Material 2

## Data Availability

The datasets generated and/or analysed during the current study are not publicly available due to the conditions and agreements under which they were collected.

## Abbreviations

NSSI: Non-suicidal self-injury
BPD: Borderline personality disorder
DBT: Dialectical behaviour therapy
KSADS: Schedule for Affective Disorders and Schizophrenia for School-Aged Children
SSRI: Selective serotonin reuptake inhibitors
C: GAS-Children’s Global Assessment Scale
