# Supplementary material for: Psychotropic medication use among adolescents participating in three randomized trials of DBT
Source: Borderline Personal Disord Emot Dysregul. 2024 Feb 22;11:5. doi: 10.1186/s40479-024-00249-0 (PMC10885477; doi:10.1186/s40479-024-00249-0)
Supplement: Supplementary file 1 — Supplementary Material 1 [file 40479_2024_249_MOESM1_ESM.docx]

| Supplemental Table 1. Baseline use of psychotropic medications by DSM-IV diagnosis across samples of the three published randomized controlled trials of DBT-A for suicidal and self-harming adolescents with borderline features | | | | | | | | | | | | |
| --- | --- | --- | --- | --- | --- | --- | --- | --- | --- | --- | --- | --- |
|  |  |  |  |  |  |  |  |  |  |  |  |  |
| Drug class | MD | | BPD | | Any anxiety disorder | | PTSD | | MD + BPD | | No MD or BPD | |
|  |  | |  | |  | |  | |  | |  | |
|  | n | % | n | % | n | % | n | % | n | % | n | % |
| The US sample (N = 173) | 142 | 82.1 | 92 | 53.2 | 93 | 54.1 | 78 | 45.1 | 79 | 45.7 | 18 | 10.4 |
| Antidepressant | 82 | 57.7 | 52 | 56.5 | 58 | 62.4 | 45 | 57.7 | 42 | 53.2 | 12 | 66.7 |
| Anxiolytic | 12 | 8.5 | 8 | 8.7 | 11 | 11.8 | 6 | 7.7 | 8 | 10.1 | 1 | 5.6 |
| Antipsychotic | 28 | 19.7 | 21 | 22.8 | 20 | 21.5 | 17 | 21.8 | 17 | 21.5 | 4 | 22.2 |
| Mood stabilizer (Lithium and/or anticonvulsants) | 25 | 17.6 | 11 | 12.0 | 19 | 20.4 | 11 | 14.1 | 11 | 13.9 | 4 | 22.2 |
| CNS stimulant | 13 | 9.2 | 10 | 10.9 | 8 | 8.6 | 7 | 9.0 | 9 | 11.4 | 3 | 16.7 |
| Antihistamine | 8 | 5.6 | 4 | 4.4 | 7 | 7.5 | 3 | 3.9 | 4 | 5.1 | 0 | 0.0 |
| Other psychotropic medications | 2 | 1.4 | 2 | 2.2 | 2 | 2.2 | 1 | 1.3 | 2 | 2.5 | 1 | 5.6 |
| Any psychotropic medication | 92 | 64.8 | 59 | 64.1 | 64 | 68.8 | 53 | 68.0 | 49 | 62.0 | 14 | 77.8 |
| No psychotropic medication | 50 | 35.2 | 33 | 35.9 | 29 | 31.2 | 25 | 32.1 | 30 | 38.0 | 4 | 22.2 |
|  |  |  |  |  |  |  |  |  |  |  |  |  |
| The Oslo sample (N = 77) | 38 | 49.4 | 15 | 20.5 | 33 | 42.9 | 13 | 16.9 | 11 | 15.1 | 27 | 37.0 |
| Antidepressant | 2 | 5.3 | 0 | 0 | 2 | 6.1 | 1 | 7.7 | 0 | 0 | 1 | 3.7 |
| Anxiolytic | 0 | 0 | 0 | 0 | 0 | 0 | 0 | 0 | 0 | 0 | 0 | 0 |
| Antipsychotic | 1 | 2.6 | 1 | 6.7 | 1 | 3.0 | 0 | 0 | 1 | 3.7 | 0 | 0 |
| Mood stabilizer | 0 | 0 | 0 | 0 | 0 | 0 | 0 | 0 | 0 | 0 | 0 | 0 |
| CNS stimulant | 1 | 2.6 | 0 | 0 | 0 | 0 | 0 | 0 | 0 | 0 | 0 | 0 |
| Antihistaminic | 2 | 5.3 | 1 | 6.7 | 0 | 0 | 1 | 7.7 | 1 | 3.7 | 0 | 0 |
| Other psychotropic medications | 0 | 0 | 0 | 0 | 0 | 0 | 0 | 0 | - | - | 1 | 3.7 |
| Any psychotropic medications | 6 | 15.8 | 2 | 13.3 | 3 | 9.1 | 1 | 7.7 | 2 | 18.2 | 2 | 7.4 |
| No psychotropic medications | 32 | 84.2 | 13 | 86.7 | 30 | 90.9 | 12 | 92.3 | 9 | 81.8 | 25 | 92.6 |
|  |  |  |  |  |  |  |  |  |  |  |  |  |
| The Barcelona sample (N = 35) | 29 | 82.9 | - | - | 19 | 54.3 | 8 | 22.8 | - | - | - | - |
| Antidepressant | 21 | 72.4 | - | - | 9 | 47.3 | 5 | 62.5 | - | - | - | - |
| Anxiolytic | 5 | 17.2 | - | - | 4 | 21 | 2 | 25 | - | - | - | - |
| Antipsychotic | 21 | 72.4 | - | - | 12 | 63.1 | 8 | 100 | - | - | - | - |
| Mood stabilizer (Lithium and/or anticonvulsants) | 4 | 13.8 | - | - | 3 | 15.8 | 2 | 25 | - | - | - | - |
| CNS stimulant | 5 | 17.2 | - | - | 2 | 10.5 | 0 | 0 | - | - | - | - |
| Antihistamine | - | - | - | - | - | - | - | - | - | - | - | - |
| Other psychotropic medications | 1 | 3.4 | - | - | 0 | 0 | 2 | 25 | - | - | - | - |
| Any psychotropic medications | 25 | 86.2 | - | - | 0 | 0 | 0 | 0 | - | - | - | - |
| No psychotropic medications | 4 | 13.8 | - | - | 3 | 15.8 | 0 | 0 | - | - | - | - |
| Notes: MD = major depression; PTSD = post-traumatic stress disorder; BPD = borderline personality disorder; "- " denotes non-available information. | | | | | | | | | | | | |
|  |  |  |  |  |  |  |  |  |  |  |  |  |
