# Supplementary material for: Psychotropic medication use among adolescents participating in three randomized trials of DBT
Source: Borderline Personal Disord Emot Dysregul. 2024 Feb 22;11:5. doi: 10.1186/s40479-024-00249-0 (PMC10885477; doi:10.1186/s40479-024-00249-0)
Supplement: Supplementary file 2 — Supplementary Material 2 [file 40479_2024_249_MOESM2_ESM.docx]

| Supplemental Table 2. Inclusion and exclusion criteria in the three randomized controlled trials of DBT-A for suicidal and self-harming adolescents with borderline features | | | | | | | |
| --- | --- | --- | --- | --- | --- | --- | --- |
|  | | US sample | | Oslo sample | | | Barcelona sample |
| *Inclusion criteria* | |  | |  | | |  |
| - Age (years) | | 12 - 18 | | 12 - 18 | | | 12 - 18 |
| - Suicide attempts (n) | | ≥ 1 during  life-time | |  | | |  |
| - Suicidal ideation | | SIQ-Jr  ≥ 24  past month | | - | | | - |
| - Self-harm (n) | | ≥ 3 life-time episodes, of which 1 during last 12 weeks | | ≥ 2 life-time episodes, of which 1 during last 16 weeks | | | ≥ 2 episodes during last 12 months |
| - BPD criteria (n) | | ≥ 3 criteria  fulfilled | | ≥ 3 criteria fulfilled | | | - |
| - Language | | Fluency in English | | Fluency in Norwegian | | | - |
| - Informed assent/consent | | Parents and adolescent | | Parents and adolescent | | | Parents and adolescent |
| *Exclusion criteria* |  | |  | | |  | |
| *- Diagnoses and conditions* | Primary problem of psychosis incl. mania, anorexia, life-threatening condition,  IQ<70 | | Psychosis incl. bipolar I, Asperger, substance or eating disorder requiring intensive treatment, IQ<70 | | Acute psycho-pathology requiring inpatient treatment, low weight anorexia, substance dependence, IQ<70 | | |
| Notes: SIQ-Jr - *Suicidal Ideation Questionnaire- Jr*; BPD - Borderline Personality Disorder. | | | | | | | |
